# Modelling of Blood Lactate Time-Courses During Exercise and/or the Subsequent Recovery: Limitations and Few Perspectives

**DOI:** 10.3389/fphys.2021.702252

**Published:** 2021-10-21

**Authors:** Rémi Durand, Mayeul Galli, Marie Chenavard, David Bandiera, Hubert Freund, Laurent A. Messonnier

**Affiliations:** Laboratoire Interuniversitaire de Biologie de la Motricité, Université Savoie Mont Blanc, Chambéry, France

**Keywords:** modelling, lactate, exercise, recovery, curves, velocity constants, kinetics

## Abstract

Because lactate is an important metabolic intermediate and a signalling molecule between/within cells/organs, it appears essential to be able to describe the kinetics of this central molecule, during and/or after physical exercise. The present study aimed to confront three models and their approaches [Freund and co-workers (F&co), Beneke and co-workers (B&co), and Quittmann and co-workers (Q&co)] to investigate the lactate exchange (γ_1_) and removal (γ_2_) abilities (min^−1^) during and/or after exercise. Nine healthy male subjects performed 3- and 6-min easy, moderate, and heavy exercise. Blood lactate concentration (BLC) was measured every 5 s over the entire period of exercise and recovery. Approaches differ depending on the domain in which the model is applied: considering exercise and part of the recovery (B&co and Q&co) or the entire period of recovery (F&co). The different approaches result in differing γ_1_ and γ_2_ values. Model fitting is closer to the experimental values following the method (model and approach) of F&co. Complementary analyses show that consideration of (i) exercise drastically impairs the quality of model fitting and therefore the γ_1_ and γ_2_ values and (ii) the entire period of recovery considerably improves the quality of fits and therefore of the γ_1_ and γ_2_ values. We conclude that (i) it is neither realistic nor reliable to take into account exercise and recovery in the same model and (ii) the longer the period of recovery studied, the better the quality of the γ_1_ and γ_2_ values.

## Introduction

Muscle contraction requires energy that comes from adenosine triphosphate (ATP) hydrolysis. Because of extremely low levels of ATP stores, several metabolic pathways are activated to resynthesize it. Glycogenolysis and glycolysis are major components of these metabolic pathways. Their activation induces lactate production, which increases with exercise intensity (Stanley et al., 1985; MacRae et al., 1992). When this production cannot be balanced anymore by removal processes, lactate accumulates in muscle (Chwalbinska-Moneta et al., 1989; Juel et al., 1990) and blood (Stanley et al., 1985; MacRae et al., 1992; Bergman et al., 1999; Messonnier et al., 2013). Blood lactate profiles obtained during incremental (Davis et al., 1983; Heck et al., 1985) or constant load exercises (Heck et al., 1985; Beneke, 1995) have been well-described. Different indexes drawn from the blood lactate vs. work rate curve have shown to be closely related to performance in different sporting activities (Faude et al., 2009) or markers of exercise intensity during endurance training in athletes (Yu et al., 2012; Tran et al., 2014) and in patients (Messonnier et al., 2021).

Lactate is well-known as a metabolic intermediate between and within cells and organs (lactate shuttle concept) for cell fuelling (Brooks, 1986, 2000, 2009) inducing and contributing to metabolic flexibility of various cells, such as cancer cells (Brooks, 2018; Brooks et al., 2021). It can also serve as a cell-signalling molecule and has the potential for the regulation of gene expression and epigenetic modifications (Hashimoto et al., 2007; Brooks, 2018; Brooks et al., 2021). From that point of view, it appears essential to be able to describe the kinetics of this central molecule, during and/or after physical exercise.

Tracer studies constitute the reference method to determine lactate kinetics parameters, such as the lactate rates of appearance (R_a_) and disappearance (R_d_) (Brooks et al., 1991; Miller et al., 2002; Messonnier et al., 2013). However, to be fully applicable and reliable, tracer techniques require almost steady states in blood lactate and tracer concentrations and a near equilibrium in concentrations between the compartments of the body (e.g., between active muscles and blood). These requirements are not fulfilled during high-intensity exercise and its subsequent recovery. Therefore, alternative methods should be found and applied. Freund and co-workers (F&Co) described the blood lactate profiles during recovery following short high-intensity exercises using a biexponential time function referring to a two-compartment lactate distribution space (Freund and Gendry, 1978; Freund and Zouloumian, 1981a,b; Zouloumian and Freund, 1981a,b). The interest of this approach lies in the fact that it allows for the determination of two major components of lactate kinetics, namely, the lactate exchange (γ_1_) and removal (γ_2_) abilities during recovery (Freund et al., 1986; Chatel et al., 2016). Of note, γ_1_ and γ_2_ should not be mixed up with R_a_ and R_d_, which refer to different concepts. Using this approach, it has been shown that lactate exchange and removal abilities decrease with exercise duration and intensity (Freund et al., 1986, 1989; Chatel et al., 2016). Therefore, these abilities refer to the physiological state of the subject at the end of the exercise. Furthermore, it has been shown that these abilities were (i) improved by endurance training (Messonnier et al., 2001), (ii) different according to physical ability profiles of athletes (Bret et al., 2003), and (iii) related to performance (Messonnier et al., 1997, 2002). Applications of this model have been proposed, allowing for the estimation of net lactate release rate, the net amount of lactate released, lactate disappearance rate, and lactate metabolic clearance rate during recovery (Bret et al., 2003; Messonnier et al., 2006; Chatel et al., 2016).

The model proposed by Freund and Zouloumian and used later by their successors involved a passive and almost complete recovery, meaning that measurements of blood lactate concentrations (BLCs) during recovery may last 60–90 min if the exercise was heavy. To bypass this inconvenience, Beneke and co-workers (B&co) (Beneke et al., 2005) and more recently Quittmann and co-workers (Q&co) (Quittmann et al., 2018) proposed adapted models and approaches allowing reductions of lactate collection time during recovery to 20 and 10 min, respectively. To obtain realistic predictions and especially a return to lifelike resting blood lactate values, their models start at the pre-exercise BLC and use only one amplitude. If this approach forces the models to return to realistic resting BLC (i.e., the pre-exercise BLC), this implies that their models apply to both exercise and recovery. While the approach proposed by F&co provides information concerning lactate exchange and removal abilities during recovery, the approaches proposed by Beneke et al. (2005) and Quittmann et al. (2018) suggest that the same lactate exchange and removal abilities prevail during both exercise and recovery. This contradicts the fact that these abilities are altered by exercise itself (Freund et al., 1986, 1989). Does it constitute a pitfall?

The aim of the present study was to assess the three approaches using the same set of experimental data and compare the results. The hypothesis is that the adapted models are less precise than the models and approaches used by F&co and less universally applicable regardless of the performed exercise.

## Materials and Methods

The data set used in the present study had been obtained previously (Freund et al., 1989). The methods are repeated here for the convenience of the reader. The study was conducted according to the laws and standards at the time of the experiment.

### Subjects

Nine healthy male subjects volunteered to participate in this study. Their age, weight, height, maximal oxygen uptake (VO_2max_), and maximal aerobic power (power associated with VO_2max_) were 21.0 ± 2.4 years old, 67.7 ± 6.4 kg, 175 ± 8 cm, 3.76 ± 0.51 L min^−1^, and 284 ± 33 W, respectively. Prior to giving their written consent, all subjects were informed of the aim and potential risks or discomforts associated with the experiments.

### Experimental Design

All exercise tests were performed on the same ergo cycle (Fleisch ergometer) at a constant pedalling frequency of 60 rpm. Prior to the experiments, subjects underwent a physical examination and an incremental exercise up to exhaustion to determine the maximal oxygen uptake (VO_2max_) and the corresponding maximal aerobic power. On two occasions 2 weeks apart, subjects were tested in the morning 2 h after having had a light standard breakfast. Under local anaesthesia, an indwelling catheter was placed in the brachial artery. The tests were performed at the normal room temperature (21–23°C). Each experiment was performed in the following order: a rest of 30 min, an easy exercise intensity [(mean ± SEM) 1.81 ± 0.03 W·kg^−1^; 39–50% VO_2max_] followed by a recovery of at least 30 min, a moderate exercise intensity (2.53 ± 0.08 W·kg^−1^; 52–67% VO_2max_) with subsequent recovery of at least 60 min, and finally, a heavy exercise intensity (3.52 ± 0.17 W·kg^−1^; 76–82% VO_2max_) followed by a recovery period of at least 90 min. On the first occasion, five subjects were randomly assigned to 3-min exercises while the four remaining subjects performed 6-min exercises. On the second occasion (2 weeks later), subjects reiterated the protocol on a crossover basis, switching from 3- to 6-min or to 6- to 3-min exercises. Each exercise and its subsequent recovery were considered separately. Seven curves were missing (three after 3-min exercises and four after 6-min exercise) due to exercises not being performed.

### Arterial Blood Sampling and Analysis

To avoid coagulation, the subjects were heparinized (100 IU·kg^−1^ body mass). Throughout exercise and recovery, arterial blood was sampled via a catheter at a rate of 0.32 mL·min^−1^ by means of a well-calibrated peristaltic pump. Arterial blood was analysed automatically for lactate concentration (Freund, 1967). Lactate concentration was determined on a continuous flow custom-made analyser using an enzymatic method where the oxidation of lactate to pyruvate is coupled in the presence of lactate dehydrogenase and NAD^+^ to NADH + H^+^ formation. The operations required for the biochemical reactions (such as mixing, dialyses, heating and additions of buffers, lactate dehydrogenase, and NAD^+^) were carried out automatically by the analyser. The changes of absorption were measured at 340 or 365 nm (5–10 nm light path). The electrical signals supplied by the colorimeter were converted in lactate concentrations by means of the two standard curves determined before and after each experiment. It is worth mentioning that the time necessary for the blood to flow through the catheter was precisely measured before each experiment. The response time of the analyser was also rigorously measured during its calibration. Both these times were accounted for the synchronisation of data with the experimental protocol. For further details, we refer the reader to previous publications (Freund, 1967; Freund et al., 1989). Total blood loss during a test amounted to 0.100–0.150 L. Signals from the biochemical analyser allowed recording of BLCs every 5 s. Experimental values of maximal blood lactate concentrations (BLC_max_, mmol L^−1^) and time to reach BLC_max_ (TBLC_max_, min) were recorded.

### Mathematical Analysis

Arterial blood lactate (exercise and/or recovery) curves were fitted by Equation 1 (Freund and co-workers), Equation 2 (Beneke and co-workers), and Equation 3 (Quittmann and co-workers) using an iterative non-linear regression technique (Kaleidagraph 3.6, Synergy Software, PA, USA).

For the convenience of the readers, the original equations have been reworded to contain consistent terminology over the three equations.


(1) $$\left[La](t)={\left[La\right]}_{\left(\text{completion}\right)}+A_{1}(1-e^{-\gamma_{\text{1}}.\text{t}})+A_{2}(1-e^{-\gamma_{2}.\text{t}})\;(1\right)$$

(2) $$\left[La](t)=[(A^{.}\gamma_{\text{1}})/(\gamma_{2}-\gamma_{1})].(e^{-\gamma_{\text{1}}.\text{t}}-e^{-\gamma_{\text{2}}.\text{t}})+{\left[La\right]}_{\left(0\right)}\;(2\right)$$

(3) $$\begin{array}{l} \left[La\right]\left(t\right)={\left[La\right]}_{\left(0\right)}+A\left(1-e^{-\gamma_{1}.\text{t}}\right)-A\left(1-e^{-\gamma_{2}.\text{t}}\right) \end{array}$$

where [La] is arterial lactate concentration (mmol·L^−1^), La_(completion)_ is the BLC at exercise completion (onset of recovery, mmol·L^−1^), [La]_(0)_ is the BLC at the beginning of exercise (resting value, mmol·L^−1^), *t* is time (min), A_1_ and A_2_ are the amplitudes of the experimental terms (mmol·L^−1^) describing lactate appearance and disappearance, respectively, A is this amplitude of appearance and disappearance (difference from maximal and resting BLC, mmol·L^−1^), and γ_1_ and γ_2_ are the velocity constants (min^−1^) describing lactate appearance and disappearance, respectively. These two latter terms, common to the three approaches, are important because they represent dynamic dimensions describing the blood lactate kinetics. BLC_max_ and TBLC_max_ values have been calculated from the parameters of the fits.

During the first series of experiments (Series 1), the three equations were applied over the domain proposed by the respective authors: only during the recovery for F&co, the starting value of physical exercise and the recovery values over 20 or 10 min for B&co or Q&co, respectively. In other words, during this first series, the three equations were applied as performed by Freund et al. (1989), Beneke et al. (2005), and Quittmann et al. (2018).

During the second series of experiments (Series 2), the three equations were fitted to the experimental BLC curves over the entire period of recovery, e.g., the starting value of physical exercise for Equations 2, 3.

During the third series of experiments (Series 3), the equations of F&co were applied to three periods of recovery: to the first 10 min, to the first 20 min, and to the entire period of the recovery.

### Statistical Analysis

Values are presented as means and SDs. Statistical analysis was processed using Jasp^®^ software (JASP Team, Version 0.12.2, Amsterdam, the Netherlands). After verification of variable homogeneity and normality by the Shapiro-Wilk test, a sphericity test was performed to compare the means using repeated-measures ANOVA. When the data respected sphericity, Holm's *post-hoc* test was applied. Otherwise, a Greenhouse-Keizer correction factor was applied. If the data did not follow normality, a row ANOVA was performed, with the application of a *post-hoc* Conover test. Correlations between sets of data have been performed. Statistical significance was set at *p* < 0.05.

## Results

### Series 1

**Figure 1:**
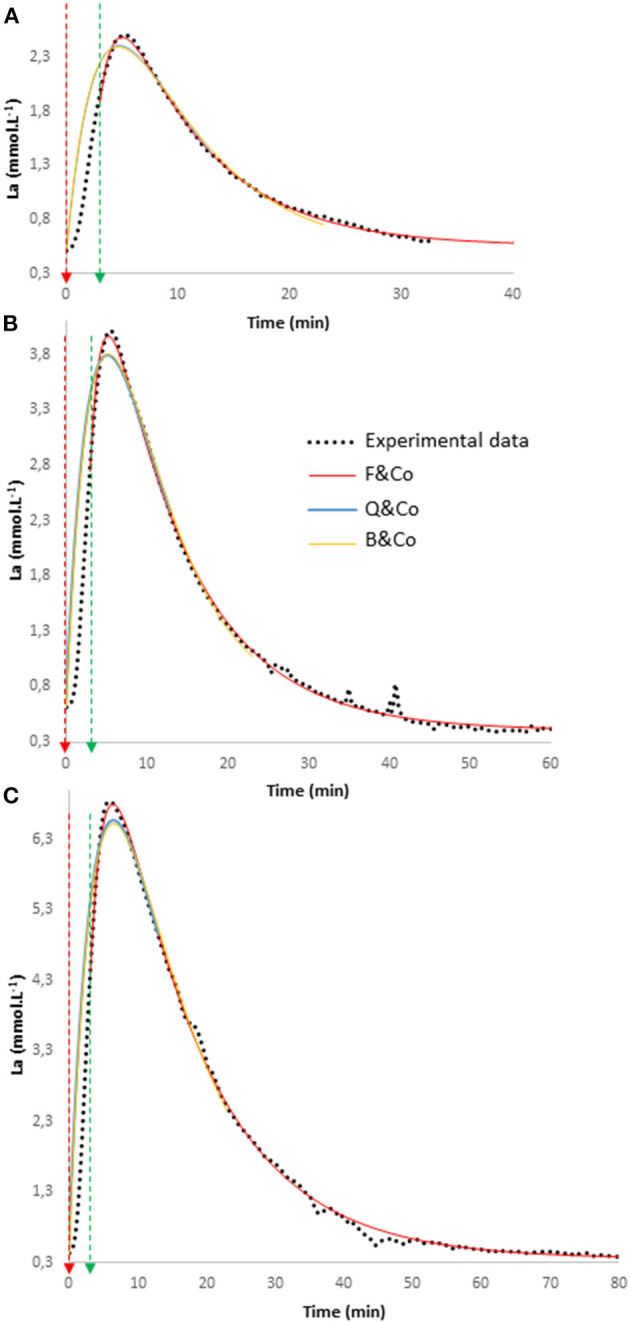
Three experimental blood lactate concentration (La) curves obtained in the same subject during 3-min exercises and the following recoveries at three different exercise intensities [easy **(A)**, moderate **(B)**, and heavy **(C)**, respectively] and the fits from Equations 1–3 applied as Freund and co-workers (F&co), Beneke and co-workers (B&co), and Quittmann and co-workers (Q&co) did (see methods).

Figure 1 reports three experimental curves of BLCs obtained in the same subject during 3-min exercises and the following recoveries at three different exercise intensities (easy, moderate, and heavy, respectively), and the model fits from Equations 1–3 following the initial recommendations of F&co, B&co, and Q&co (see methods). Graphically, it can be observed that applying a model during the recovery only (F&co method) describes more closely the recovery experimental values rather than taking into account the pre-exercise value and part of the recovery (B&co or Q&co methods).

Table 1 reports the mean values of the velocity constants (γ_1_ and γ_2_) along with the R^2^ values of the fits on the experimental data. The repeated measures of ANOVAs show significant differences between the three approaches (*p* < 0.001) for all the considered parameters. The *post hoc* tests highlight significant differences between the data obtained by F&co and B&co (*p* < 0.001), and F&co and Q&co (*p* < 0.001). The mean values of γ_1_ and γ_2_ are not different between the methods applied by B&co and Q&co (Table 1). The coefficients of determination R^2^ show significant differences (*p* < 0.001) between the three approaches, the R^2^ obtained by F&co being the highest. Parameters obtained with the different approaches are on the other hand significantly correlated (Table 1). By focusing only on the 3- and 6-min heavy intensity exercises, all parameters are different (Table 1), and more importantly, correlations between parameters are not obvious. In that sense, it seems that the longer the exercise, the weaker the correlations (Table 1).

**Table 1:** Mean values of the velocity constants γ_1_ and γ_2_ along with the R^2^ values of the fits on the experimental data of Freund et al., 1989.

	**Mean ± SD**			**ANOVA** **(*p* value)**	***Post-hoc*** **(*****p*** **value)**			**Correlation (*****r*** **value**, ***p*** **value)**		
	**F&co**	**B&co**	**Q&co**		**F&co vs. B&co**	**F&co vs. Q&co**	**B&co vs. Q&co**	**F&co vs. B&co**	**F&co vs. Q&co**	**B&co vs. Q&co**
*n* = 38, 3, or 6 min at easy, moderate, or heavy-intensity exercises										
γ_1_ (min^−1^)	0.973 ± 0.641	0.320 ± 0.215	0.264 ± 0.131	**<0.001**	**<0.001**	**<0.001**	0.916	0.84, **<0.001**	0.75, **<0.001**	0.87, **<0.001**
γ_2_ (min^−1^)	0.0947 ± 0.0289	0.1273 ± 0.0425	0.1475 ±0.0579	**<0.001**	**<0.001**	**<0.001**	0.362	0.71, **<0.001**	0.84, **<0.001**	0.72, **<0.001**
R^2^	0.999 ±0.001	0.993 ± 0.005	0.974 ± 0.021	**<0.001**	**<0.001**	**<0.001**	**<0.001**			
*n* = 8, 3 min heavy intensity exercises										
γ_1_ (min^−1^)	0.721 ± 0.217	0.241 ± 0.080	0.173 ± 0.024	**<0.001**	**<0.001**	**<0.001**	**0.05**	0.86, **0.006**	0.67, 0.064	0.82, **0.013**
γ_2_ (min^−1^)	0.0742 ± 0.0155	0.1125 ± 0.0250	0.1517 ± 0.0212	**<0.001**	**0.012**	**<0.001**	0.062	0.26, 0.535	0.69, 0.058	0.30, 0.475
R^2^	0.999 ± 0.001	0.988 ± 0.004	0.950 ± 0.016	**<0.001**	0.065	**0.001**	0.065			
*n* = 7, 6 min heavy intensity exercises										
γ_1_ (min^−1^)	0.434 ± 0.298	0.209 ± 0.058	0.261 ± 0.038	**0.017**	**0.012**	0.086	0.306	0.79, 0.035	0.04, 0.929	0.04, 0.928
γ_2_ (min^−1^)	0.0634 ± 0.0084	0.0800 ± 0.0168	0.0587 ± 0.0184	**0.003**	**0.004**	0.326	**0.046**	0.78, 0.037	0.33, 0.465	0.22, 0.635
R^2^	0.999 ± 0.001	0.993 ± 0.001	0.983 ± 0.005	**<0.001**	**<0.001**	**<0.001**	**<0.001**			

*The three equations were applied over the domain proposed by the respective authors, namely, only during the recovery for Freund and co-workers (F&co), the starting value of physical exercise and the recovery values over 20 or 10 min for Beneke and co-workers (B&co) or Quittmann and co-workers (Q&co), respectively. p values ≤ 0.05 are reported in bold*.

Table 2 reports the mean values recorded on the experimental data for BLC_max_ and TBLC_max_ and those obtained from the fits. As it can be seen in Table 2, BLC_max_ and TBLC_max_ are underestimated by the approaches of B&co and Q&co.

**Table 2:** Mean values of maximal blood lactate concentration (BLC_max_) and time to reach BLC_max_ (TBLC_max_) obtained from the experimental data (ExpData) or predicted by the fits, some of the parameters (γ_1_ and γ_2_) being reported in Table 1.

	**ExpData**	**F&co**	**B&co**	**Q&co**	**F&co vs. ExpData**	**B&co vs. ExpData**	**Q&co vs. ExpData**
*n* = 38, 3, or 6 min at easy, moderate, or heavy-intensity exercises							
BLC_max_ (mmol.L^−1^)	5.46 ± 2.80	5.42 ± 2.78	5.31 ± 2.71	5.31 ± 2.70	0.072	**<0.001**	**<0.001**
TBLC_max_ (min)	2.06 ± 0.88	1.87 ± 0.84	1.30 ± 1.70	1.53 ± 1.46	0.072	**0.011**	**0.012**
*n* = 8, 3 min heavy-intensity exercises							
BLC_max_ (mmol.L^−1^)	7.20 ± 1.10	7.13 ± 1.14	6.83 ± 1.10	6.89 ± 1.09	0.455	**<0.001**	**0.015**
TBLC_max_ (min)	2.84 ± 0.55	2.80 ± 0.62	3.14 ± 0.79	3.27 ± 0.80	0.831	0.339	0.241
*n* = 7, 6 min heavy-intensity exercises							
BLC_max_ (mmol.L^−1^)	9.48 ± 1.38	9.41 ± 1.38	9.30 ± 1.34	9.25 ± 1.32	0.217	**0.015**	**0.009**
TBLC_max_ (min)	2.39 ± 1.00	2.36 ± 0.71	1.93 ± 1.05	1.67 ± 1.01	0.921	0.465	0.167

*p values ≤ 0.05 are reported in bold*.

### Series 2

**Figure 2:**
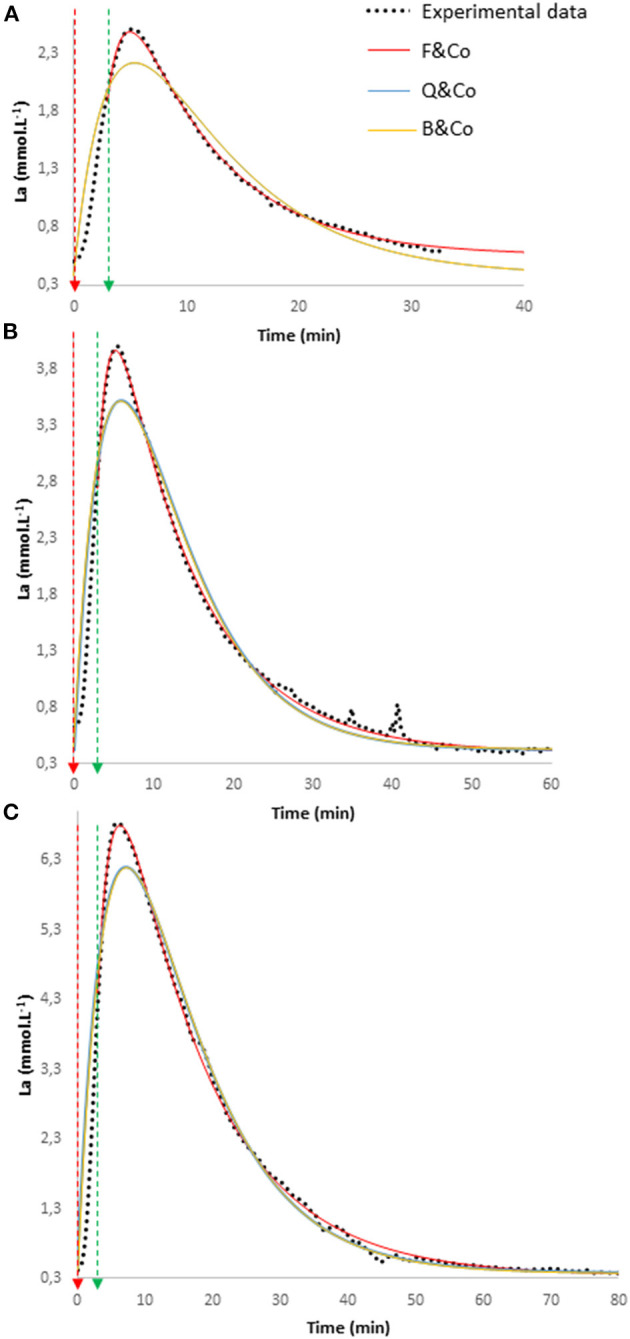
Three experimental blood lactate concentration (La) curves obtained in the same subject during 3-min exercises and the following recoveries at three different exercise intensities [easy **(A)**, moderate **(B)**, and heavy **(C)**, respectively] and the fits from Equations 1–3 applied over the entire period of recovery (for F&co) and taking also into account the initial blood lactate concentration at the beginning of exercise (for B&co and Q&co).

Figure 2 reports three curves obtained in the same subject during 3-min exercises and the following recoveries at three different exercise intensities (easy, moderate, and heavy, respectively) applying the three approaches over the entire period of recovery. Graphically, it can be observed that applying a model during the recovery only (F&co) describes more closely the experimental values than taking into account the pre-exercise value and part of the recovery (B&co or Q&co).

Table 3 reports the mean values of the velocity constants, γ_1_ and γ_2_, along with the R^2^ values of the fits on the experimental data. The repeated measures of ANOVA show significant differences between the three approaches (*p* < 0.001) for all the considered parameters. The *post-hoc* tests highlight significant differences between the data obtained by F&co and B&co (*p* < 0.001), and F&co and Q&co (*p* < 0.001). The mean values of γ_1_ and γ_2_ are not different between the methods applied by B&co and Q&co (Table 3). The coefficients of determination R^2^ show significant differences (*p* < 0.001) between the three approaches, the R^2^ obtained by the method of F&co being the highest. Parameters obtained with the different approaches are on the other hand significantly correlated (Table 3).

**Table 3:** Mean values of the velocity constants γ_1_ and γ_2_ along with the R^2^ values of the fits on the experimental data of Freund et al. (1989).

	**Mean ± SD**			**ANOVA (*p* value)**	***Post-hoc*** **(*****p*** **value)**			**Correlation (*****r*** **value**, ***p*** **value)**		
	**F&co**	**B&co**	**Q&co**		**F&co vs. B&co**	**F&co vs. Q&co**	**B&co vs. Q&co**	**F&co vs. B&co**	**F&co vs. Q&co**	**B&co vs. Q&co**
*n* = 41, 3, or 6 min at easy, moderate, or heavy-intensity exercises										
γ_1_ (min^−1^)	0.960 ± 0.628	0.210 ± 0.068	0.214 ± 0.066	**<0.001**	**<0.001**	**<0.001**	0.117	0.58, **<0.001**	0.62, **<0.001**	0.99, **<0.001**
γ_2_ (min^−1^)	0.0984 ± 0.0403	0.1324 ± 0.0526	0.1280 ± 0.0490	**<0.001**	**<0.001**	**<0.001**	0.08	0.79, **<0.001**	0.81, **<0.001**	0.99, **<0.001**
R^2^	0.998 ± 0.003	0.978 ± 0.010	0.978 ± 0.010	**<0.001**	**<0.001**	**<0.001**	0.118			
*n* = 8, 3 min heavy intensity exercises										
γ_1_ (min^−1^)	0.721 ± 0.217	0.222 ± 0.046	0.223 ± 0.044	**0.041**	**0.002**	0.156	**0.05**	0.47, 0.240	0.56, 0.149	0.99, **<0.001**
γ_2_ (min^−1^)	0.0742 ± 0.0155	0.0977 ± 0.0349	0.0962 ± 0.0313	**0.009**	**0.001**	**0.002**	0.779	0.99, **<0.001**	0.99, **<0.001**	0.99, **<0.001**
R^2^	0.999 ± 0.001	0.984 ± 0.005	0.984 ± 0.005	**<0.001**	**0.003**	**0.003**	1.00			
*n* = 7, 6 min heavy intensity exercises										
γ_1_ (min^−1^)	0.434 ± 0.298	0.138 ± 0.035	0.141 ± 0.032	**0.002**	**0.005**	0.054	0.206	0.81, **0.028**	0.83, **0.022**	0.99, **<0.001**
γ_2_ (min^−1^)	0.0634 ± 0.0084	0.0905 ± 0.0218	0.0874 ± 0.0189	**0.002**	**<0.001**	**<0.001**	0.453	0.93, **0.003**	0.95, **0.001**	0.99, **<0.001**
R^2^	0.999 ± 0.001	0.989 ± 0.003	0.989 ± 0.003	**<0.001**	**<0.001**	**<0.001**	0.997			

*The three equations were applied over the entire period of recovery for Freund and co-workers (F&co) and taking into account the starting value of physical exercise for Beneke and co-workers (B&co) or Quittmann and co-workers (Q&co), respectively. p values ≤ 0.05 are reported in bold*.

Table 4 reports the mean values recorded on the experimental data for BLC_max_ and TBLC_max_ and those obtained from the fits. As it can be seen in Table 4, BLC_max_ and TBLC_max_ are underestimated and overestimated, respectively, by the approaches of B&co and Q&co.

**Table 4:** Mean values of maximal blood lactate concentration (BLC_max_) and time to reach BLC_max_ (TBLC_max_) obtained from the experimental data (ExpData) or predicted by the fits, some of the parameters (γ_1_ and γ_2_) being reported in Table 2.

	**ExpData**	**F&co**	**B&co**	**Q&co**	**F&co vs. ExpData**	**B&co vs. ExpData**	**Q&co vs. ExpData**
*n* = 41, 3, or 6 min at easy, moderate, or heavy-intensity exercises						
BLC_max_ (mmol.L^−1^)	5.44 ± 2.75	5.40 ± 2.73	4.81 ± 2.48	4.83 ± 2.52	0.13	**<0.001**	**<0.001**
TBLC_max_ (min)	2.08 ± 0.84	1.92 ± 0.82	2.46 ± 1.16	2.45 ± 1.16	0.3	**0.022**	**0.023**
*n* = 8, 3 min heavy-intensity exercises						
BLC_max_ (mmol.L^−1^)	7.20 ± 1.10	7.13 ± 1.14	6.37 ± 1.12	6.37 ± 1.12	0.455	**0.004**	**0.002**
TBLC_max_ (min)	2.84 ± 0.55	2.80 ± 0.62	3.79 ± 0.63	3.78 ± 0.63	1.00	**<0.001**	**<0.001**
*n* = 7, 6 min heavy-intensity exercises						
BLC_max_ (mmol.L^−1^)	9.48 ± 1.38	9.41 ± 1.38	8.43 ± 1.34	8.63 ± 1.45	0.565	**<0.001**	**<0.001**
TBLC_max_ (min)	2.39 ± 1.10	2.36 ± 0.71	3.09 ±0.73	3.09 ±0.73	1.00	0.64	0.064

*p values ≤ 0.05 are reported in bold*.

### Series 3

Table 5 reports the mean γ_1_ and γ_2_ values using the model of Freund et al. applied on the entire period of recovery, or only during the first 20 or 10 min of recovery. γ_1_ decreases significantly with the shortening of the recovery duration taken into account. On the contrary, γ_2_ increases significantly with the shortening of the recovery duration taken into account. Taking into account only the heavy exercises (of 3 or 6 min), the γ_1_ and γ_2_ values are not different but they are not correlated to each other, meaning that hierarchisation of results/subjects on the basis of their γ_1_ and γ_2_ values is drastically obscured.

**Table 5:** Mean values of the velocity constants γ_1_ and γ_2_ along with the R^2^ values of the fits on the experimental data of Freund et al. (1989).

	**Mean ± SD**			**ANOVA (*p* value)**	***Post-hoc*** **(*****p*** **value)**			**Correlation (*****r*** **value**, ***p*** **value)**		
	**F&co**	**F&co20'**	**F&co10'**		**F&co vs. F&co20'**	**F&co vs. F&co10'**	**F&co20' vs. F&co10'**	**F&co vs. F&co20'**	**F&co vs. F&co10'**	**F&co20' vs. F&co10'**
*n* = 40, 3, or 6 min at easy, moderate, or heavy-intensity exercises										
γ_1_ (min^−1^)	0.956 ± 0.633	0.724 ± 0.250	0.664 ± 0.380	**<0.001**	**0.001**	**<0.001**	0.504	0.85, **<0.001**	0.64, **<0.001**	0.76, **<0.001**
γ_2_ (min^−1^)	0.0966 ± 0.0339	0.1166 ± 0.1057	0.1475 ± 0.0511	**0.007**	**0.021**	**0.003**	0.504	0.80, **<0.001**	0.76, **<0.001**	0.83, **<0.001**
R^2^	0.999 ± 0.001	0.998 ± 0.004	0.997 ± 0.001	**<0.001**	**0.007**	**0.016**	**<0.001**			
*n* = 8, 3 min heavy-intensity exercises										
γ_1_ (min^−1^)	0.721 ± 0.217	0.593 ± 0.132	0.591 ± 0.099	0.121	/	/	/	0.619, 0.170	0.052, 0.904	0.245, 0.558
γ_2_ (min^−1^)	0.0742 ± 0.0155	0.0836 ± 0.0280	0.0901 ± 0.0862	0.687	/	/	/	0.532, 0.175	**0.753, 0.031**	0.854, **0.007**
R^2^	0.999 ± 0.001	0.999 ± 0.001	0.994 ± 0.005	**0.002**	1.00	**0.01**	**0.01**			
*n* = 7, 6 min heavy-intensity exercises										
γ_1_ (min^−1^)	0.473 ± 0.298	0.473 ± 0.347	0.658 ± 0.289	0.156	/	/	/	0.113, 0.810	0.088, 0.851	0.845, **0.017**
γ_2_ (min^−1^)	0.0634 ± 0.0084	0.0571 ± 0.0350	0.0467 ± 0.0095	0.156	/	/	/	0.373, 0.411	0.106, 0.822	0.324, 0.478
R^2^	0.999 ± 0.001	0.999 ± 0.001	0.993 ± 0.006	**0.018**	0.603	**0.02**	0.054			

*The model of Freund and co-workers was applied on the entire period of recovery (F&co), or taking into account only 20 min (F&co20') or 10 min (F&co10') or recovery. p values ≤ 0.05 are reported in bold*.

## Discussion

The present study aimed to test different models and approaches in depicting and interpreting blood lactate curves during unsteady-state exercise and/or the subsequent recovery. As a whole, it appears that applying models and approaches (i) during exercise and recovery or (ii) during recovery but only over a restricted part of it present pitfalls. In these conditions, the models do not fit adequately to the experimental curves, challenging the interpretation of the velocity constants describing lactate appearance in and disappearance from the blood. Applying models and approaches only during the recovery and over the entire period of recovery (until returning to resting blood lactate values) still appears as the most accurate way to determine the lactate exchange and removal abilities.

### Different Approaches and Different Results

Considering the mean values of parameters obtained by Beneke et al. (2005) after application of their model over the exercise and 20 min of recovery in response to a 0.5-min Wingate test, a return to the near resting BLCs after 200 min of recovery can be predicted. This time-to-return for resting blood lactate values is unrealistic. For instance, this duration is much longer (by ~1 h) than the one (~140 min) predicted from data obtained by Maciejewski et al. (2013) using the F&co model applied on the entire period of recovery after 3-min all-out exercises, while the exercise was totally exhaustive and led to higher BLCs than in the former case (Beneke et al., 2005). This first comparison already casts doubt on the fact that the different models and approaches provide congruent results. To answer the question more clearly, our first analysis compared the different parameters obtained after fitting the models on the same experimental set of data (Freund et al., 1989).

In the conditions of applying the equation proposed by each group (i.e., over recovery for F&co, over exercise, and 20 or 10 min of recovery for B&co or Q&co, respectively), the values of the velocity constants are drastically different (Table 1). Figure 1 shows clearly that the equation proposed by F&co fits almost perfectly the recovery curve. On the other hand, the fits from B&co and Q&co equations are more often aside from the experimental data. The deviations are particularly important in the initial phase of the recovery, rendering the determination of the peak BLC during the recovery difficult with the two latter approaches. To illustrate this latter point, BLC_max_ and TBLC_max_ are different from the experimental values using the approaches of B&co and Q&co, but are not different using the approach of F&co (Table 2).

One may argue that even if the results are different, correlations still exist between parameters obtained from the different approaches (Table 1). However, it is important to keep in mind that blood lactate curves have been obtained in very different conditions (3- or 6-min, easy-, moderate-, or heavy-intensity exercises). Therefore, the question arises as to whether the correlations are still observable for a given exercise, rendering hierarchisation between subjects possible and accurate. Table 3 reports stratified data according to the performed exercises. As it can be seen, only very few correlations between parameters obtained using the different approaches are obtained.

From that point of view, the lactate exchange and removal abilities (namely, γ_1_ and γ_2_) obtained from the three approaches are not identical. Two main differences exist between the approaches of F&co, and B&co and Q&co. The two differences lie (i) on the consideration of exercise and recovery or just recovery and (ii) on the considered period of recovery (partial or complete recovery). Therefore, are the differences observed between the approaches related to the first, the second, or a combination of the two differences? The following analyses will try to answer the question.

### Effect of Exercise on Blood Lactate Kinetics Parameters (Series 2)

Comparison of data obtained using the three equations on the entire period of recovery (and taking into account the starting exercise value for B&co and Q&co) allows to apprehend the effect of taking into account the exercise on the parameter values. Table 3 reports the data obtained using this paradigm and underlines very different γ_1_ and γ_2_ values between the approaches used by F&co and both those used by B&co and Q&co. The fits are also closer to the experimental data using the F&co approach as indicated by the R^2^ values and the correspondence with BLC_max_ and TBLC_max_ (Table 4; Figure 2). Together, these results suggest that considering exercise is detrimental for good fitting and determination of the parameters of the blood lactate recovery kinetics. Actually, this is not so surprising, previous studies have shown that γ_1_ and γ_2_ values decrease with exercise duration and intensity (Freund et al., 1986, 1989). Consistent with the decrease of γ_1_ as the exercise progresses, Juel et al. (1990) observed that during ~3-min maximal exercises, the net lactate release rate from the active muscle to the blood increased and then tended to plateau, suggesting that lactate exchanges between active muscles and blood are disturbed when maximal exercise progresses. Therefore, if γ_1_ and γ_2_ are apparently constant during recovery, it appears unrealistic to apply the same constant γ_1_ and γ_2_ values for exercise and recovery.

Another way to demonstrate the limits of taking into account exercise and recovery is that approaches, such as exercise and recovery, cannot be applied during and after long-lasting (i.e., 60 min) exercises, while the approach proposed by Freund and co-workers is still valuable (Freund et al., 1990).

### Effect of Length of Considered Period During Recovery

We also suspect that the length of the considered period of recovery is critical for an accurate determination of γ_1_ and γ_2_. A first clue tending to confirm this inference was the lower R^2^ values obtained using the approach of Q&co (who considered only the initial 10 min of recovery) compared to the one used by B&co (who considered the first 20 min of recovery). To further investigate the importance of the length of the considered recovery period, we performed two additional analyses. First, a comparison was performed between the data obtained in Series 1 (considering only 20 or 10 min of recovery) and Series 2 (considering the entire period of recovery) for 3- and 6-min heavy-intensity exercises using the approaches of B&co and Q&co (Tables 1, 3). According to the period of recovery taken into account, values of parameters changed. Second, a comparison was performed between the data obtained after exercise using the F&co approach applied (i) on the full length, (ii) over 20 min, or (iii) over 10 min of recovery (Series 3; Table 5). Clearly, γ_1_ and γ_2_ values are different according to the length of recovery taken into account (Table 5). The shorter the recovery, the lower the γ_1_ and the higher the γ_2_. These complementary analyses indicate that it is better to take into account the entire recovery period. Actually, as it can be seen in Figures for moderate or heavy exercise, there are important inflexion points on the curves beyond the 20 min of recovery. These points need to be taken into account for precise γ_2_ determinations.

### Limitations

In the present report, we criticised the fact that taking into account exercise and recovery are not very precise in so far as the dynamical parameters that drive the blood lactate curve (according to the models depicted here), namely, the lactate exchange and removal abilities, change during exercise. In that regard, applying constant values during exercise and recovery makes little sense. Actually, the same criticism could be made to the F&co model and approach. Indeed, the probability that γ_1_ and γ_2_ remain constant over the entire period of recovery is very low. Indeed, if it is obvious that at the onset of the recovery, γ_1_ and γ_2_ reflect the physiological state of the subject at that time, it is also obvious that at the end of the recovery, γ_1_ and γ_2_ are almost returned to resting conditions. Nevertheless, the fits of the model are very close to the experimental data (R^2^ > 0.999) so that everything appears as if the lactate exchange and removal abilities remain constant all over the recovery.

### Perspectives

Although theoretically not perfect, the model and approach proposed by F&co seem for the moment the most accurate to describe the blood lactate curve during recovery and determine accurately two important aspects of its kinetics: the lactate exchange and removal abilities (namely, γ_1_ and γ_2_, respectively). Therefore, we recommend using this model and its domain of application for the entire period of recovery.

In the present study, the models and approaches proposed by B&co and Q&co have been applied on a set of data obtained during and after 3- or 6-min exercises. In such circumstances, strong limitations of these models and approaches to describe the blood lactate curve and thus to accurately determine γ_1_ and γ_2_ appeared. However, we acknowledge that the impact of exercise on the fits and on the determination of γ_1_ and γ_2_ would have been much lower if very short exercises (typically 15 or 30 s) had been used. Therefore, we strongly encourage the future scholars who would like to use the B&co or Q&co models and approaches to strictly apply these models (i) only during and after very short exercises and importantly (ii) on the entire period of recovery. Indeed, whatever the model and approach are, a precise determination of the lactate removal ability (γ_2_) requires a long period of recovery.

## Conclusion

Taken together, caution should be taken concerning the interpretation of the velocity constants obtained from the models and approaches proposed by Beneke et al. (2005) and Quittmann et al. (2018) if exercise is longer than 30 s and if the recovery is incomplete. At present, it seems that the model and approach proposed by F&co, although tedious due to the long period of recovery studied, remains the most accurate way to describe the blood lactate recovery curve and determine the lactate exchange and removal abilities during recovery.

## Data Availability Statement

The data analyzed in this study is subject to the following licences/restrictions: Data have been previously published. Original data have been obtained from the authors. Requests to access these datasets should be directed to Hubert Freund, hubert.freund@wanadoo.fr.

## Ethics Statement

We reused data already published. At the time of the experiments, the study complied with the law of the country for studies involving human participants and with the declaration of Helsinki. The patients/participants provided their written informed consent to participate in this study.

## Author Contributions

LM and HF designed and conducted this study and wrote the first draft of the article. RD, MG, MC, and DB analyzed data. All authors critically reviewed the draft and approved the final version for publication.

## Conflict of Interest

LM receives consulting fees from Abbott Diabetes Care. All are outside the submitted work. The remaining authors declare that the research was conducted in the absence of any commercial or financial relationships that could be construed as a potential conflict of interest.

## Publisher's Note

All claims expressed in this article are solely those of the authors and do not necessarily represent those of their affiliated organizations, or those of the publisher, the editors and the reviewers. Any product that may be evaluated in this article, or claim that may be made by its manufacturer, is not guaranteed or endorsed by the publisher.
